# Co_3_O_4_ Nanopetals Grown on the Porous CuO Network for the Photocatalytic Degradation

**DOI:** 10.3390/nano12162850

**Published:** 2022-08-18

**Authors:** Yuntao Sun, Can Wang, Shengyao Qin, Fengda Pan, Yongyan Li, Zhifeng Wang, Chunling Qin

**Affiliations:** 1 School of Materials Science and Engineering, Hebei University of Technology, Tianjin 300401, China; 2 Key Laboratory for New Type of Functional Materials in Hebei Province, Hebei University of Technology, Tianjin 300401, China

**Keywords:** nanoporous, nanopetals, Co_3_O_4_@NP-CuO, heterojunction, photocatalytic

## Abstract

Designing a novel photocatalytic composite for the efficient degradation of organic dyes remains a serious challenge. Herein, the multi-layered Co_3_O_4_@NP-CuO photocatalyst with unique features, i.e., the self-supporting, hierarchical porous network as well as the construction of heterojunction between Co_3_O_4_ and CuO, are synthesized by dealloying-electrodeposition and subsequent thermal treatment techniques. It is found that the interwoven ultrathin Co_3_O_4_ nanopetals evenly grow on the nanoporous CuO network (Co_3_O_4_@NP-CuO). The three-dimensional (3D) hierarchical porous structure for the catalyst provides more surface area to act as active sites and facilitates the absorption of visible light in the photodegradation reaction. Compared with the commercial CuO and Co_3_O_4_ powders, the newly designed Co_3_O_4_@NP-CuO composite exhibits superior photodegradation performance for RhB. The enhanced performance is mainly due to the construction of heterojunction of Co_3_O_4_/CuO, greatly promoting the efficient carrier separation for photocatalysis. Furthermore, the possible photocatalytic mechanism is analyzed in detail. This work provides a promising strategy for the fabrication of a new controllable heterojunction to improve photocatalytic activity.

## 1. Introduction

Water is an essential natural resource for life, inseparable from human health and the sustainability of economic development. Due to the rapid development of industrialization, most of the freshwater resources have been consumed, resulting in a large amount of wastewater contaminated with organic compounds, heavy metal ions, and dyes, which has a serious impact on human production and life. Dye wastewater (from hair dyes, leather, textiles, and solar luminescence technology), one of the most common pollutants, is highly toxic and carcinogenic [1,2,3,4,5,6,7]. Nowadays, a series of strategies are adopted to address environmental issues, such as biodegradation technology [8], physical adsorption [9], and advanced oxidation processes (AOPs) [10,11,12]. Especially, the AOPs, with the generation of strongly oxidizing hydroxyl radicals (·OH) and superoxide radicals (·O_2_^−^) in the catalytic reactions, have attracted considerable attention due to the ability of rapid organic decomposition and efficient wastewater purification. Photocatalytic semiconductor degradation, one of the AOPs, has many advantages, including no secondary pollution, low expenses, and complete oxidation of organic dyes, etc. [13,14].

As far, a p-type semiconductor Co_3_O_4_ (the band gap of ~1.7–2.3 eV) [15,16,17], with the different morphologies of nanoparticles [18], nanosheets [19], and quantum dots [20], has been utilized in electrochemical sensors, electrodes, and magnetic materials, respectively. Considering the theoretical good visible light response range of Co_3_O_4_ [21], it is promising in the field of photocatalytic degradation. Hence, it is of great importance to study the photocatalytic degradation properties of Co_3_O_4_.

Unfortunately, numerous semiconductors (including Co_3_O_4_) [22,23,24,25] encounter a major problem of narrow optical response range, which results in high photogenerated-carriers recombination rate. Among the variety of modifications [26,27], the construction of heterojunction/Schottky junction by epitaxial growth can efficiently facilitate spatial charge separation and further improve the photocatalytic performance, as exemplified for Co_3_O_4_/TiO_2_ [28], Cu_x_O/ZnO@Au [29] and Co_3_O_4_/g-C_3_N_4_ [30]. Another issue is how to reduce the agglomeration of powders-based composite photocatalysts obtained by conventional preparations [31,32]. To better solve the above-mentioned structural and functional drawbacks, it is necessary to design a new type of photocatalyst simultaneously possessing a self-supporting structure and heterojunction or Schottky junction structure.

Recently, nanoporous metals (NPMs) and metallic composites have remarkable features in the field of catalysis on account of fertile active sites exposed in the unique three-dimensional (3D) nanoporous structure [33,34,35,36,37,38,39], attracting much attention in the photocatalysis field. For instance, Wang et al. [40] reported a high degradable activity of 3D nanostructure rutile TiO_2_ photocatalyst by dealloying Cu_60_Ti_30_Y_10_ amorphous in HNO_3_ aqueous solutions. In our previous work, Cu_2_O/CuO nanowires/nanosheets [41,42] and Au nanoparticles modified CuO nanowires grew on nanoporous Cu (NPC) [43] have been successfully synthesized via a simple dealloying-anodizing technique and the composites exhibited much-improved degradation performance.

The work in this paper synthesized a novel Co_3_O_4_@CuO composite catalyst by utilizing the dealloying Cu_40_Zr_60_ amorphous alloy followed by the electrodeposition methods [44] along with heat treatment. The Co_3_O_4_@CuO composite exhibits an intriguing multi-layered structure and concurrently possesses the self-supporting, hierarchical porous network as well as the Co_3_O_4_/CuO heterojunction, which not only avoids the agglomeration of powder-like catalysts but also accelerates the transport of photogenerated carriers and reduces the recombination of electrons (e^−^) and holes (h^+^). The photodegradable performance in rhodamine b (RhB) was further measured. Moreover, the underlying degradation mechanism in the photocatalytic process was analyzed in detail by using active radicals capture experiments.

## 2. Materials and Methods

### 2.1. Synthesis of Nanoporous Cu (NPC)

Alloy ingots of Cu_40_Zr_60_ (at. %) were prepared by arc-melting a mixture of pure Cu (99.99 wt%) and Zr (99.99 wt%) under a Zr-gettered argon atmosphere. The Cu_40_Zr_60_ amorphous ribbons with a width of 2 mm and a thickness of ~25 μm were fabricated by remelting and quenching onto a rotating copper wheel at a linear speed of 34 m/s [45]. Then, the nanoporous Cu (NPC) was fabricated by free-chemical dealloying the as-spun Cu_40_Zr_60_ ribbons immersed in 0.05 M HF solution for 2 h at 298 K [43]. The as-dealloyed ribbons (NPC) were cleaned with deionized water and dried at 333 K in a vacuum.

### 2.2. Synthesis of Co_3_O_4_@NP-CuO Composite

The as-dealloyed ribbons (NPC, with a length of 35 mm) were directly used as the deposition substrate network. Then, the Co(OH)_2_ nanopetals were electrodeposited on the NPC substrate network (Co(OH)_2_@NPC) in the 60 mM Co(NO_3_)_2_·6H_2_O via the electrodeposition method [44], where NPC served as the working electrode, a platinum network as a counter electrode, and Ag/AgCl as a reference electrode. The electrochemical deposition was performed at −0.8 V for 30 min. The as-calcined ribbons (Co_3_O_4_@NP-CuO composite) were further obtained by calcining the as-deposited ribbons (Co(OH)_2_@NPC) at 573 K for 2 h in the air.

### 2.3. Microstructure Characterization

The phase and crystal structure of the prepared samples were characterized by an X-ray diffractometer (XRD, D8, Cu-Kα, Bruker, Karlsruhe, Germany). The chemical valences of elements were detected using an X-ray photoelectron spectrometer (XPS, Thermo Fisher Scientific, Waltham, MA, USA). Transmission electron microscopy (TEM, JEOL JEM 2100F, Tokyo, Japan) and scanning electron microscopy (SEM, Nova nanoSEM 450, FEI, Hillsboro, OR, USA) were applied to characterize the microstructure of the samples. The photoluminescence spectra (PL) were detected on a fluorescence spectrometer (FL3-22). The ASAP 2020M+C type-specific surface area and porosity analyzer were used to measure specific surface area and the pore diameter distribution of the samples via BET and BJH methods, respectively.

### 2.4. Degradation Experiments

The simultaneous degradation experiments were carried out under the irradiation of a xenon lamp light source (100 mW/cm^2^, λ ≥ 420 nm) to study the degradation performance of photocatalysts. During the experiments, the catalyst with an effective catalytic area of 1 cm^2^ (ca 2 mg) was added into the mixed 6 mL of 10 mg/L RhB solution and 2 mL of 30 mass% H_2_O_2_. The concentration of RhB was measured on a UV-vis spectrophotometer (UV-1920) every 2 min. Tert-butanol (TBA), p-benzoquinone (PBQ), silver nitrate (AgNO_3_), and disodium ethylenediaminetetraacetate (EDTA-2Na) with a consistent amount (2 mL of 1 mM) were added to the above solutions to capture hydroxyl radicals (·OH), superoxide radicals (·O_2_^−^), electrons (e^−^) and holes (h^+^), respectively to identify the essential active species. The active species were further determined by the electron paramagnetic resonance (EPR) using Bruker A300 electron paramagnetic resonance spectrometer.

## 3. Results and Discussion

### 3.1. Design of Self-Supporting Co_3_O_4_@NP-CuO Composite

**Figure 1:**
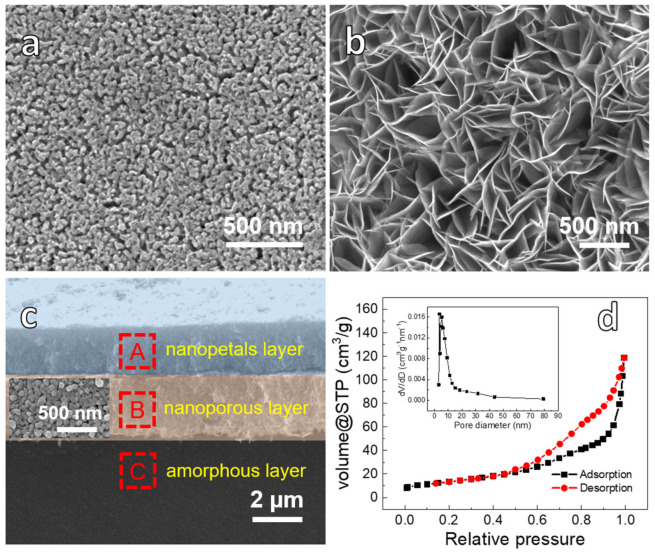
(**a**) SEM images of Cu_40_Zr_60_ amorphous alloy ribbon de-alloyed in 0.05 M HF for 2 h; The composite sample via deposition at −0.8 V for 30 min followed by the calcination: (**b**) Plan-view SEM images; (**c**) Cross-sectional SEM image; (**d**) Nitrogen adsorption-desorption curve and pore size distribution of Co_3_O_4_@NP-CuO.

Figure 1a shows the SEM image of the as-dealloyed ribbon (NPC) obtained by dealloying Cu_40_Zr_60_ ribbons in 0.05 M HF solutions for 2 h. It is clearly seen that the as-dealloyed ribbon exhibits a 3D bicontinuous pore/ligament structure [43]. The as-dealloyed ribbon is chosen as the deposition substrate network. First, to explore the effect of deposition potential on the surface morphology grown on the as-dealloyed ribbon substrate, Figure S1 shows that SEM images of the as-deposited ribbons after electrodeposition in 60 mM cobalt nitrate solutions, at applied potentials of −0.6, −0.8, −1.0, and −1.2 V for 30 min, respectively. Unlike the uneven distribution of nanopetals (Figure S1a) and the coarsen nanopetals (Figure S1c,d), it is observed that the most uniform and fine nanopetals grow on the NPC substrate (Figure S1b) when the deposited voltage is at −0.8 V. Thus, the electrodeposition voltage and time of −0.8 V and 30 min, respectively, are selected as the following electrodeposition conditions.

After depositing (Figure S1b), the as-deposited samples are further calcined at 573 K for 2 h, shown in Figure 1b. In Figure 1b, it is clearly observed that the interwoven nanopetals uniformly grow on the as-calcined sample surface [44], which is similar to those of as-deposited samples before heat treatment.

Figure 1c displays the cross-sectional SEM image of the as-calcined ribbon. It can be clearly observed that the as-calcined ribbon exhibits a unique three-layer structure in which the interfaces are in close contact with each other [44]. Their corresponding EDS spectra are shown in Figure S2. For the nanopetals deposition layer (region A), the EDS spectrum (Figure S2a) mainly shows the peaks of Co and O with an atomic ratio of ~3:4, implying the formation of the Co_3_O_4_ nanopetals layer. The nanoporous layer (region B) is composed of Cu and O elements in Figure S2b, corresponding to the nanoporous CuO layer (NP-CuO). It should be noticed that, compared with the morphology of NPC (Figure 1a), the ligaments of nanoporous layer (the inset image of Figure 1c) become coarsened, which is caused by the calcination treatment and the oxidation of Cu substrate network, but the CuO inherits the 3D bicontinuous nanoporous structure of NPC. Furthermore, the EDS spectrum of the inter-region C (Figure S2c) consists mainly of Cu and Zr elements with an atomic ratio of ~4:6, indicating that the region belongs to the Cu_40_Zr_60_ amorphous layer. Based on the above results, it can be concluded that the Co_3_O_4_@NP-CuO composite sample, synthesized via the dealloying and electrodeposition method followed by the calcination, exhibits a multilayer structure. In addition, uniform nanoporous structures of the NPC substrate network (Figure S3a) and uniform growth of nanopetals on the NPC (Figure S3b) can be observed at a larger scale range, which guarantees the homogeneity of Co_3_O_4_/NP-CuO composite samples. Moreover, the entire composite material keeps a free-standing structure, which is attributed to the amorphous alloy interlayer support [46,47].

The nitrogen adsorption-desorption isotherms and pores size distribution curves of Co_3_O_4_@NP-CuO are shown in Figure 1d. It belongs to the type IV isotherm curve [43], indicating that the Co_3_O_4_@NP-CuO sample possesses a mesoporous structure [44,48]. The specific surface area of Co_3_O_4_@NP-CuO is about 49.58 m^2^/g. The pores size of the material (the inset of Figure 1d) is mainly in the range of 5–15 nm, which derives from the pores on the NP-CuO matrix and the pores formed by the interweaving of nanopetals in the Co_3_O_4_ layer.

**Figure 2:**
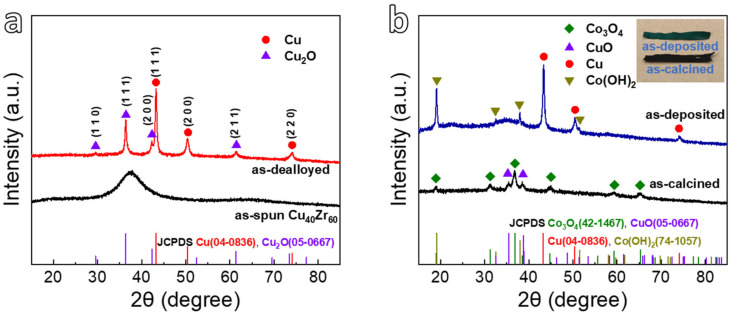
(**a**) XRD patterns of the as-spun Cu_40_Zr_60_ ribbon and as-dealloyed sample immersed in 0.05 M HF for 2 h; (**b**) XRD patterns of as-deposited sample immersed in 60 mM Co(NO_3_)_2_ at −0.8 V for 30 min and as-calcined sample at 573 K for 2 h.

The XRD patterns of all samples are shown in Figure 2. The as-spun Cu_40_Zr_60_ ribbons have a broad diffraction peak (Figure 2a), showing the formation of an amorphous structure [43,49]. After dealloying, the fcc Cu (JCPDS #04-0836) crystalline peaks (Figure 2a) are indexed in the XRD pattern of as-dealloyed ribbons, and no Zr element is detected, implying that Zr element selectively dissolves into the HF solution and the formation of nanoporous Cu (NPC) shown in Figure 1a [43]. On the other hand, the NPC substrate is selected for electrodeposition at −0.8 V for 30 min and calcination treatment at 573 K for 2 h in air. Figure 2b shows the XRD patterns of as-deposited and as-calcined ribbons. In addition to the metallic Cu peaks, the as-deposited ribbons (without calcination) show new characteristic diffraction peaks at 19.1°, 32.5°, 38.0°, and 51.5°, which are indexed as the (001), (100), (011), and (012) planes of Co(OH)_2_ (JCPDS #74-1057), respectively [44]. After calcination, the crystalline phases of the as-calcined ribbons are identified to be CuO (JCPDS #45-0937) and Co_3_O_4_ (JCPDS #42-1467), revealing the formation of nanoporous CuO (NP-CuO) on the NPC and Co(OH)_2_ nanopetals change to be Co_3_O_4_ nanopetals [50]. The results can be verified in the SEM image in Figure 1c and the EDS spectra of Figure S2.

**Figure 3:**
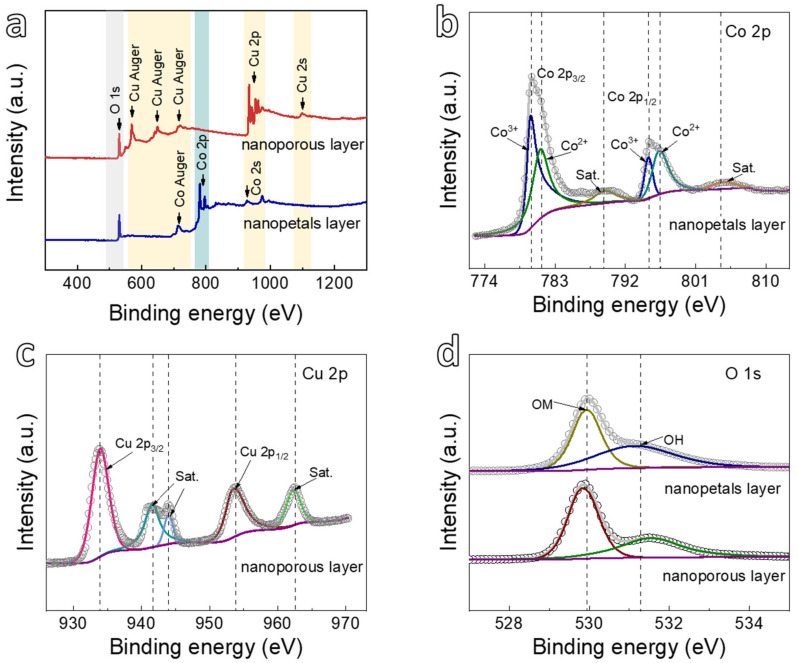
(**a**) XPS survey of the Co_3_O_4_ nanopetals layer and CuO nanoporous layer for the Co_3_O_4_@NP-CuO composite; (**b**) The deconvolution of Co 2p spectrum of the nanopetals layer; (**c**) Cu 2p spectrum of nanoporous layer; (**d**) O 1s spectra of Co_3_O_4_ nanopetals layer and CuO nanoporous layer for the Co_3_O_4_@NP-CuO.

As for the as-calcined ribbon exhibiting the multi-layer structure (Figure 1c), it is important to make clear the surface composition and chemical states of either the nanopetal layer or nanoporous layer of the as-calcined ribbon. Figure 3 shows XPS analysis applied to the nanopetal layer or nanoporous layer. From Figure 3a, the strong photoelectron peaks of Co appear in the nanopetals layer, whereas the Cu peaks significantly reduce as compared to those in the nanoporous layer. Figure 3b shows the deconvolution of the Co 2p peaks obtained from the nanopetals layer. The binding energy peaks of the Co 2p spectrum at 779.9 and 795.0 eV are related to Co 2p_3/2_ and Co 2p_1/2_ of Co^2+^ with shakeup satellite peaks at 789.4 and 804.7 eV, respectively [50]. Additionally, the two peaks centered at 781.2 and 796.4 eV are assigned to the characteristic Co 2p_3/2_ and Co 2p_1/2_ of Co^3+^, revealing the coexistence of Co^2+^ and Co^3+^ species in the Co_3_O_4_@NP-CuO [51]. Moreover, the peak intensity of Cu 2p is not obviously seen in Figure S4, which might attribute to the thicker deposited nanopetals layer. On the other hand, for the nanoporous layer just beneath the nanopetals layer, the XPS survey spectrum of the nanoporous layer (Figure 3a) identifies the Cu and O elements. In Figure 3c, the peaks of Cu 2p spectra located around 934.0 and 953.7 eV correspond to Cu 2p_3/2_ and Cu 2p_1/2_ of Cu^2+^, respectively [52]. Moreover, the Zr element is not detected in the nanoporous layer, inferring that Zr has been selectively etched after dealloying the Cu_40_Zr_60_ amorphous ribbon [43]. For both the layers displayed in Figure 3d, the two peaks of O 1s at 529.9 and 531.2 eV correspond to the metal oxides (OM) and absorbed oxygen (OH), respectively [53,54,55], demonstrating that the nanopetals layer is mainly Co_3_O_4_ and the nanoporous layer is CuO.

**Figure 4:**
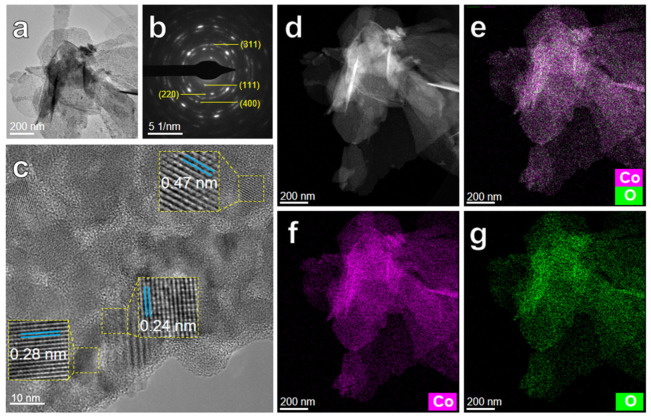
TEM images of nanopetals layer of the Co_3_O_4_@NP-CuO composite: (**a**) high magnification image; (**b**) SAED pattern; (**c**) HRTEM image; EDS mapping images of nanopetals of (**d**) dark field image; (**e**) Co and O mixed; (**f**) Co; (**g**) O.

**Figure 5:**
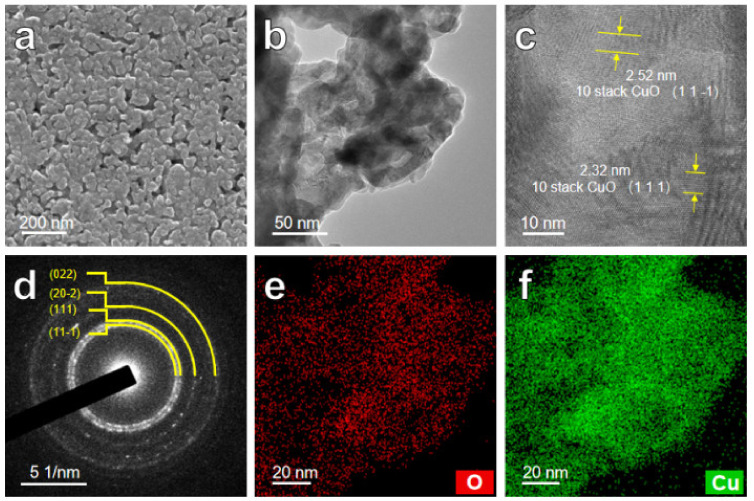
SEM and TEM images of nanoporous layer of the Co_3_O_4_@NP-CuO: (**a**) high magnification SEM image; (**b**) TEM image; (**c**) HRTEM image; (**d**) SAED pattern; EDS mapping images of (**e**) O; (**f**) Cu.

More morphologies and structure characterizations of the as-calcined multi-layered ribbon are further detected by TEM (Figure 4 and Figure 5). TEM images of the nanopetals layer for the Co_3_O_4_@NP-CuO composite are shown in Figure 4. Figure 4a shows 3D interwoven ultrathin nanopetals in the Co_3_O_4_ nanopetals layer. The SAED pattern in Figure 4b shows the (111), (220), (311), and (400) crystal orientation index, confirming the presence of the Co_3_O_4_ phase [56]. Meanwhile, from the HRTEM of nanopetals shown in Figure 4c, the lattice fringes with a spacing of 0.24 nm, 0.28 nm, and 0.47 nm are assigned to (311), (220), and (111) planes of Co_3_O_4_ (JCPDS #42-1467), respectively [51,56]. The elemental distribution of the Co_3_O_4_ nanopetals is further measured by EDS mapping, as depicted in Figure 4d–g. From the dark field image of the nanopetals shown in Figure 4d, it is clearly observed that the interwoven and superimposed structure of Co_3_O_4_ nanopetals. Obviously, Co and O elements uniformly accumulate in the Co_3_O_4_ nanopetals, revealing that the nanopetals are mainly composed of Co and O elements.

The nanopetals layer of the as-calcined ribbon is confirmed to be mainly Co_3_O_4_, while the CuO nanoporous layer needs to be more characterized. Figure 5a,b show the SEM and TEM images of the CuO nanoporous layer, respectively. It is observed that ligaments after calcination become coarsened, and the pore features are inherited. From the HRTEM of the nanoporous layer (Figure 5c), it is found that the lattice fringes spacing of 0.252 and 0.232 nm are the (11-1) and (111) of CuO, respectively [43,57]. The SAED pattern (Figure 5d) further proves the existence of the CuO phase. Moreover, the elemental mapping of the nanoporous layer is shown in Figure 5e,f. The Cu and O elements evenly distribute within the 3D ligaments, confirming the NPC is oxidized to be NP-CuO. Hence, the multi-layer porous Co_3_O_4_@NP-CuO composite with a 3D bicontinuous pore/ligament skeleton structure, self-supporting as well as the Co_3_O_4_/CuO heterojunction is successfully synthesized via the dealloying and electrodeposition methods followed by the calcination.

### 3.2. Photocatalytic Degradation Activity of Co_3_O_4_@NP-CuO Composite

**Figure 6:**
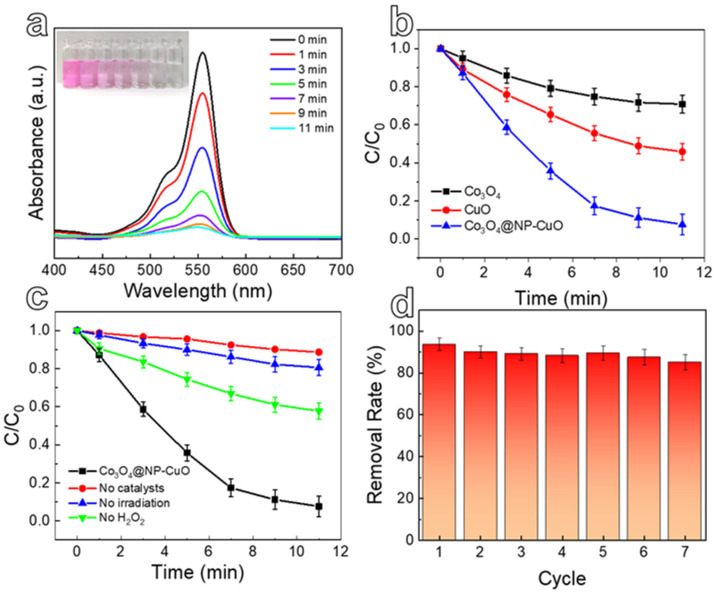
(**a**) UV-visible absorption spectra of RhB by the Co_3_O_4_@NP-CuO composite in the presence of H_2_O_2_ for different times; (**b**) degradation performance of the Co_3_O_4_@NP-CuO composite, commercial CuO, and Co_3_O_4_ powders; (**c**) effects of different conditions on degradation of RhB; (**d**) cyclic stability of Co_3_O_4_@NP-CuO composite.

The photocatalytic activity of the Co_3_O_4_@NP-CuO composite was evaluated by degrading RhB at room temperature. Figure 6a displays photocatalytic degradation results of Co_3_O_4_@NP-CuO composite in a mixed solution of RhB and H_2_O_2_ under illumination. The strong characteristic adsorption peak of RhB at 553 nm continuously decreases with time and becomes very weak after 11 min. From the inset of Figure 6a, the color change process of the RhB dye is shown, eventually becoming transparent. Figure 6b demonstrates the different degradation efficiencies of RhB by the commercial CuO, Co_3_O_4_ powders, and the composite. Compared to the commercial CuO and Co_3_O_4_, the newly designed Co_3_O_4_@NP-CuO composite exhibits an outstanding photocatalytic activity towards RhB decomposition due to the synergistic effect of the porous NP-CuO and the ultrathin Co_3_O_4_ nanopetals.

In order to explore the influence of catalyst, light-irradiation, and oxidizing agent H_2_O_2_ on the degradation performance, Figure 6c shows the performance graphs of RhB degradation in different conditions. The RhB is partially reduced in the absence of H_2_O_2_, indicating that H_2_O_2_ acts an auxiliary role in the photocatalytic process. It is worth noting that RhB is only degraded by 15–20% without a catalyst or light-irradiation. Notably, the degradation performance is significantly enhanced when all the Co_3_O_4_@NP-CuO catalyst, H_2_O_2_ and light-irradiation are applied to the RhB reaction system. The results indicate that the catalyst, H_2_O_2,_ and light-irradiation are indispensable for the rapid degradation of RhB. The synergistic effect of the catalyst, irradiation, and H_2_O_2_ will be proposed in the degradation mechanism. On the other hand, the photocatalytic degradation efficiencies, and recycling performance of RhB over the Co_3_O_4_@NP-CuO were further measured. The Co_3_O_4_@NP-CuO composite maintains a high RhB degradation rate of over 85% within seven cycles, demonstrating high recyclability.

**Figure 7:**
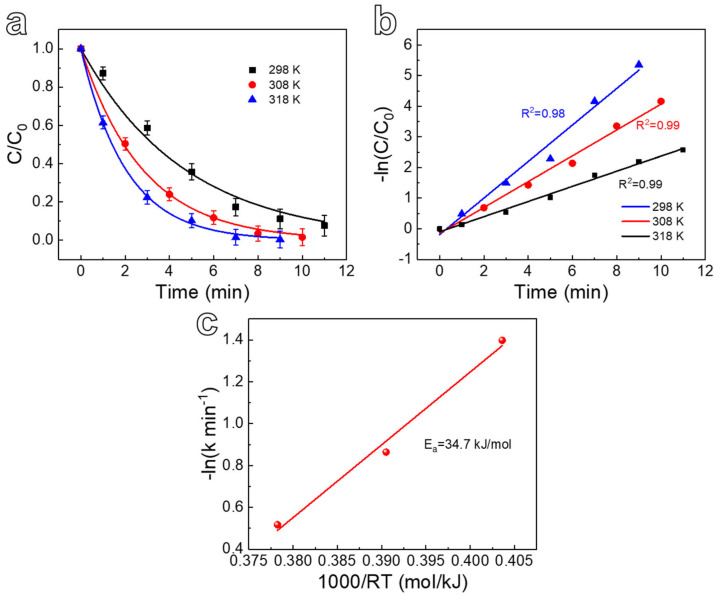
(**a**) Degradation properties of Co_3_O_4_@NP-CuO composite at different temperatures, (**b**) corresponding L-H pseudo-first-order dynamic model and, (**c**) Arrhenius plot.

The kinetic model of the photodegradation process can be well evaluated by the photodegradation rate and the activation energy of the photodegradation reaction possesses. Figure 7 clearly shows the photocatalytic degradation rate and activation energy of the Co_3_O_4_@NP-CuO composite. The performance curves of Co_3_O_4_@NP-CuO composite for RhB degradation at different temperatures are shown in Figure 7a. As the temperature of the degradation process increases from 298 K to 318 K, the degradation rate of RhB increases continuously. The degradation rate of RhB follows the pseudo-first-order kinetic model [41,42,43], as shown in Equation (1):

(1) $$\ln(\frac{C_{t}}{C_{0}})=-\mathrm{kt}$$

where C_0_ and C_t_ correspond to the transient concentration of at time 0 and at time t, respectively, k is the reaction rate constant of the photocatalytic degradation process. Combining Figure 7a with Equation (1), −ln(C_t_/C_0_) versus t for RhB photodegradation at different temperatures is drawn and fitted in Figure 7b. At different temperatures, −ln(C_t_/C_0_) shows a good linear relationship with t. The calculated photocatalytic degradation rates k at 298, 308, and 318 K are 0.25, 0.42, and 0.60 min^−1^, respectively. Furthermore, the activation energy of the photocatalytic degradation process can be evaluated according to the Arrhenius Equation (2):

(2) $$\mathrm{lnk}=-\frac{E_{a}}{\mathrm{RT}}+\mathrm{lnA}$$

In the formula, k is the photocatalytic rate constant at different temperatures, E_a_ is the apparent activation energy, R is the ideal molar gas constant, T is the thermodynamic temperature, and A is the frequency factor. The curve in Figure 7c is established with −lnk as the ordinate and 1000/RT as the abscissa. There is a good linear relationship between −lnk and 1000/RT. According to Equation (2), the activation energy of Co_3_O_4_@NP-CuO is calculated to be ~34.7 kJ/mol for the RhB degradation reaction from 298 to 318 K. The lower activation energy reflects that the photocatalytic degradation process is easier to carry out, which also proves the photocatalytic performance of the Co_3_O_4_@NP-CuO composite.

### 3.3. Degradation Mechanism

**Figure 8:**
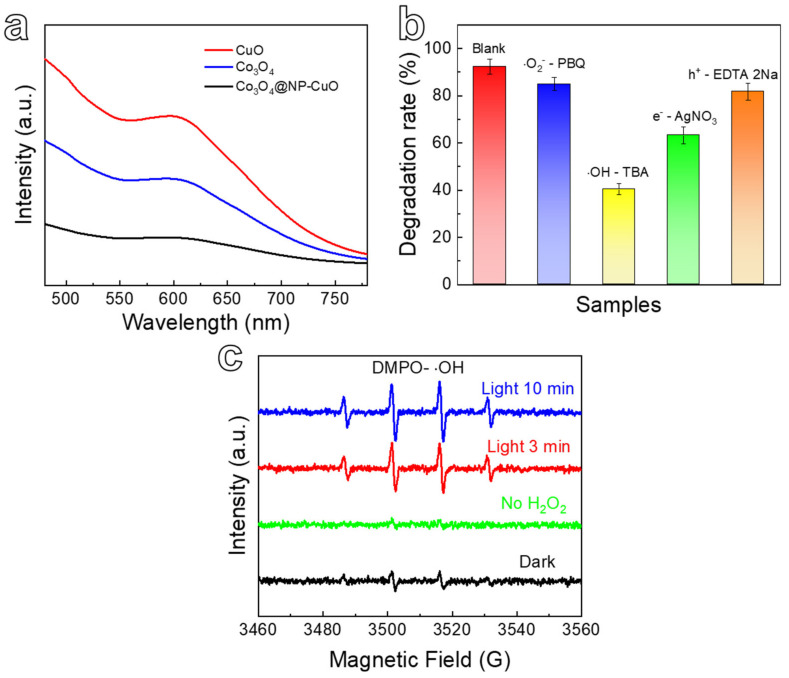
(**a**) PL spectra of Co_3_O_4_@NP-CuO, CuO, and Co_3_O_4_; (**b**) Effect of different free radicals on photocatalytic degradation of Co_3_O_4_@NP-CuO; (**c**) EPR spectra of ·OH generated under different conditions for the Co_3_O_4_@NP-CuO.

It is well-known that the recombination of photogenerated e^−^ and h^+^ for the semiconductor catalysts greatly influences the degradation efficiency. The recombination of photogenerated e^−^ and h^+^ can release energy in the form of luminescence and exotherm [41,42]. Therefore, the photoluminescence (PL) spectrum of CuO, Co_3_O_4_, and Co_3_O_4_@NP-CuO composite can be utilized to evaluate photogenerated charges recombination behavior. Figure 8a shows the PL spectra of Co_3_O_4_@NP-CuO composite, CuO and Co_3_O_4_. They exhibit different fluorescence intensities around λ = 610 nm. Compared with the fluorescence intensities of CuO and Co_3_O_4_, the fluorescence intensity of the Co_3_O_4_@NP-CuO composite is significantly lower, indicating that the Co_3_O_4_/CuO heterostructure is beneficial in reducing the recombination rate of photogenerated carriers [15,57].

In the photocatalytic degradation process, active radicals always play a significant role. To unveil the photocatalytic degradation mechanism of Co_3_O_4_@NP-CuO composite, radical trapping experiments were carried out (Figure 8b). During the photocatalytic reaction, TBA, PBQ, AgNO_3_, and ethyl EDTA-2Na were added to the RhB solution as trapping agents for OH, O^2−^, e^−^, and h^+^, respectively [43]. When the TBA is introduced, the degradation rate of RhB is apparently limited, and the degradation efficiency of the Co_3_O_4_@NP-CuO composite decreases from 92% to 40%, demonstrating that ·OH is the dominant species in the degradation of RhB. After the addition of AgNO_3__,_ the degradation rate of RhB is limited to a certain extent, and the degradation rate decreases from 92% to 63%, implying that e^−^ is also the main active species in the photocatalytic degradation process. On the contrary, the degradation rate changes slightly when PBQ and EDTA-2Na are added, inferring that ·O^2−^ and h^+^ have a minor effect on the rapid degradation reaction of RhB. Based on the above analysis, ·OH and e^−^ are the main active species in the process of RhB degradation for the present catalyst.

The electron paramagnetic resonance (EPR) technology was implemented to further disclose the generation process of free radicals (Figure 8c). It is found that a small amount of ·OH is generated in the dark, mainly due to the Fenton-like reaction of CuO and Co_3_O_4_ such as Equations (3)–(6) [41,43,58,59,60]:

(3) $${\mathrm{Cu}}^{2+}{+H}_{2}O_{2}\;\to {\;\mathrm{Cu}}^{+}+\mathrm{HOO}\cdot {+H}^{+}$$

(4) $${\mathrm{Cu}}^{+}{+H}_{2}O_{2\;}\to {\;\mathrm{Cu}}^{2+}+\cdot {\mathrm{OH}+\mathrm{OH}}^{-}$$

(5) $${\mathrm{Co}}^{2+}{+H}_{2}O_{2}\;\to {\;\mathrm{Co}}^{3+}+\cdot {\mathrm{OH}+\mathrm{OH}}^{-}$$

(6) $${\mathrm{Co}}^{3+}{+H}_{2}O_{2}\;\to {\;\mathrm{Co}}^{2+}+\mathrm{HOO}\cdot {+H}^{+}$$

The production of ·OH is greatly improved after the addition of H_2_O_2_, indicating that H_2_O_2_ is essential in the production of ·OH. Besides the Fenton-like reaction, it should be noted that the role of e^-^ cannot be ignored because H_2_O_2_ acts as an acceptor of e^-^ and react to generate ·OH (Equation (7)) [43]:

(7) $$e^{-}{+H}_{2}O_{2}\to {\mathrm{OH}}^{-}+\cdot \mathrm{OH}$$

Combined with the above results and analysis, the photocatalytic degradation mechanism of Co_3_O_4_@NP-CuO is proposed. The degradation of RhB includes the Fenton-like reaction (Equations (3)–(6)) and photocatalytic reaction (Equation (7)). As shown in Figure 6c, the photocatalytic reaction dominates the degradation process. The efficient degradation is attributed to the synergistic effect of light-irradiation and H_2_O_2_. Light and H_2_O_2_ are essential to produce ·OH [43]. Based on Equation (7), the large-scale generation of ·OH is mainly due to the reaction between photogenerated electrons produced by light-irradiation and H_2_O_2_ in Co_3_O_4_@NP-CuO. On the other hand, the 3D bicontinuous pore/ligament skeleton structure and Co_3_O_4_/CuO heterojunction together contribute to promoting photocatalytic performance [41,42,43]. The abundant Co_3_O_4_ nanopetals can absorb more light and generate more photogenerated e^−^ and h^+^. The 3D mesoporous structure of NP-CuO accelerates the transfer of photogenerated carriers, thereby effectively reducing the undesirable recombination of e^−^ and h^+^ [48]. In addition, the exposed surface of Co_3_O_4_@NP-CuO can provide more active sites for the improvement of photocatalytic degradation efficiency. Overall, the novel Co_3_O_4_@NP-CuO composite with a 3D bicontinuous pore/ligament skeleton structure exhibits excellent performance, opening a new avenue for designing photocatalytic materials.

## 4. Conclusions

In this work, the multi-layered homogenous Co_3_O_4_@NP-CuO composite simultaneously exhibiting the self-supporting, porous network as well as the Co_3_O_4_/CuO heterojunction is successfully achieved via the dealloying-electrodeposition methods followed by the calcination. The interwoven “petal-like” Co_3_O_4_ ultrathin nanosheets uniformly grow on the nanoporous CuO substrate network. The present Co_3_O_4_@NP-CuO composite exhibits much higher photodegradable performance for RhB as compared to the commercial CuO and Co_3_O_4_ powders. The rich porosity of multi-layered homogenous composite catalyst facilities the absorption of visible light and transportation of RhB dyes to generate more electrons (e^−^) and ·OH. Furthermore, the construction of heterojunction between Co_3_O_4_ and CuO effectively promotes the separation of photogenerated carriers, which is attributed to the higher performance under the synergistic effect of H_2_O_2_.

## Data Availability

The data presented in this study are available on request from the corresponding author.

## Supplementary Materials

The following supporting information can be downloaded at: https://www.mdpi.com/article/10.3390/nano12162850/s1, Figure S1: SEM images of deposition at a voltage of −0.6 V (a), −0.8 V (b), −1.0 V (c), and −1.2 V (d); Figure S2: EDS analysis for the cross-sectional image of as-calcined ribbon: nanopetals layer (a), nanoporous layer (b), amorphous layer (c); Figure S3: SEM images of a wide range of Cu_40_Zr_60_ amorphous alloy ribbon dealloyed in 0.05 M HF for 2 h (a) and the composite sample via deposition at −0.8 V for 30 min followed by the calcination (b); Figure S4: XPS spectrum of Cu 2p for nanopetals layer.
