# Supplementary material for: Co3O4 Nanopetals Grown on the Porous CuO Network for the Photocatalytic Degradation
Source: Nanomaterials (Basel). 2022 Aug 18;12(16):2850. doi: 10.3390/nano12162850 (PMC9416053; doi:10.3390/nano12162850)
Supplement: Supplementary file 1 [file nanomaterials-12-02850-s001.zip › nanomaterials-1824647-supplementary.pdf]

nanomaterials

Supporting Information

Co_3O_4 Nanopetals Grown on the Porous CuO Network for the Photocatalytic Degradation

Yuntao Sun [1], Can Wang [1], Shengyao Qin [1], Fengda Pan [1,*], Yongyan Li [1,2], Zhifeng Wang [1,2] and Chunling Qin [1,2,*]

[1] School of Materials Science and Engineering, Hebei University of Technology, Tianjin 300401, China
[2] Key Laboratory for New Type of Functional Materials in Hebei Province, Hebei University of Technology, Tianjin 300401, China
* Correspondence: 202021801120@stu.hebut.edu.cn (F.P.); clqin@hebut.edu.cn (C.Q.)

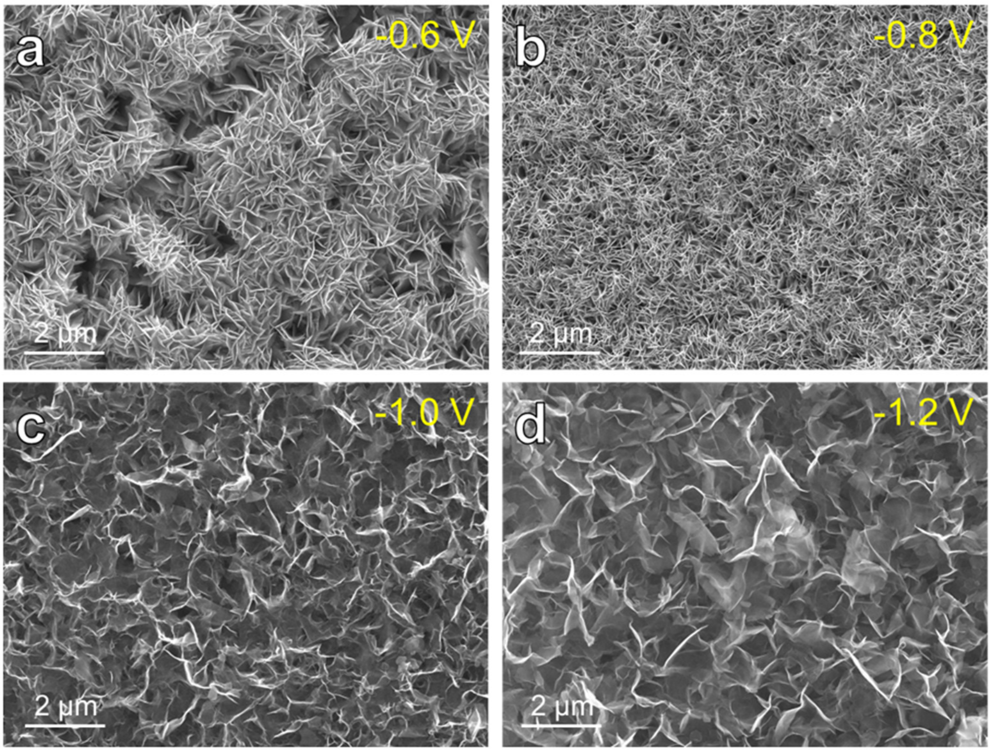

Figure S1. SEM images of deposition at voltage of -0.6 V (**a**), -0.8 V (**b**), -1.0 V (**c**) and -1.2 V (**d**).

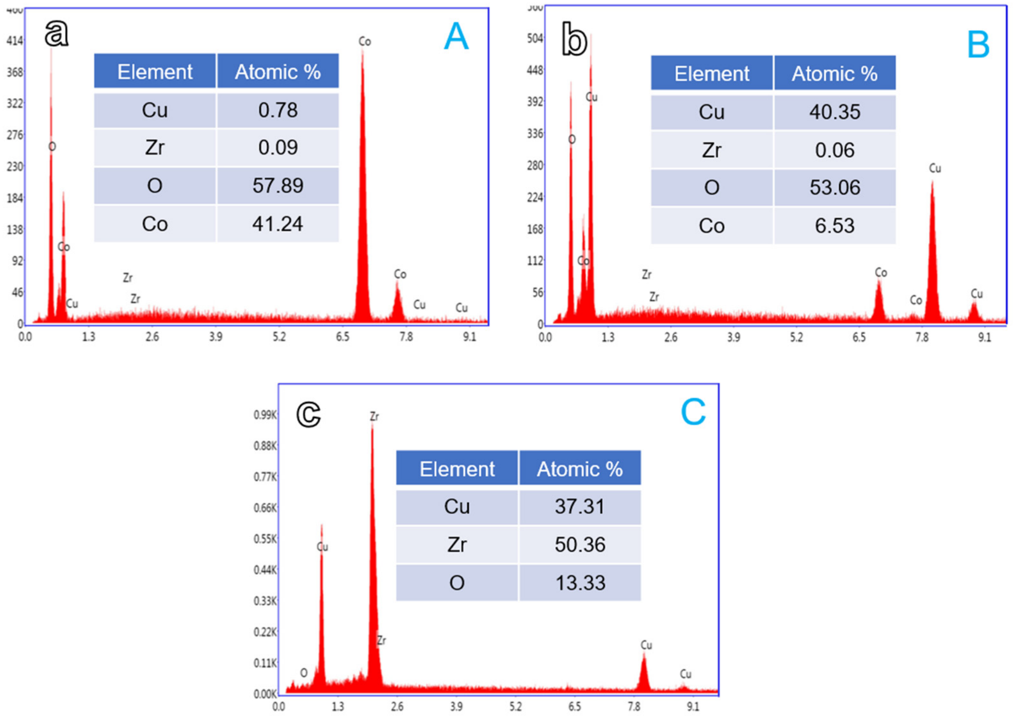

Figure S2. EDS analysis for the cross-sectional image of as-calcined ribbon: nanopetals layer (**a**), nanoporous layer (**b**), amorphous layer (**c**).

.

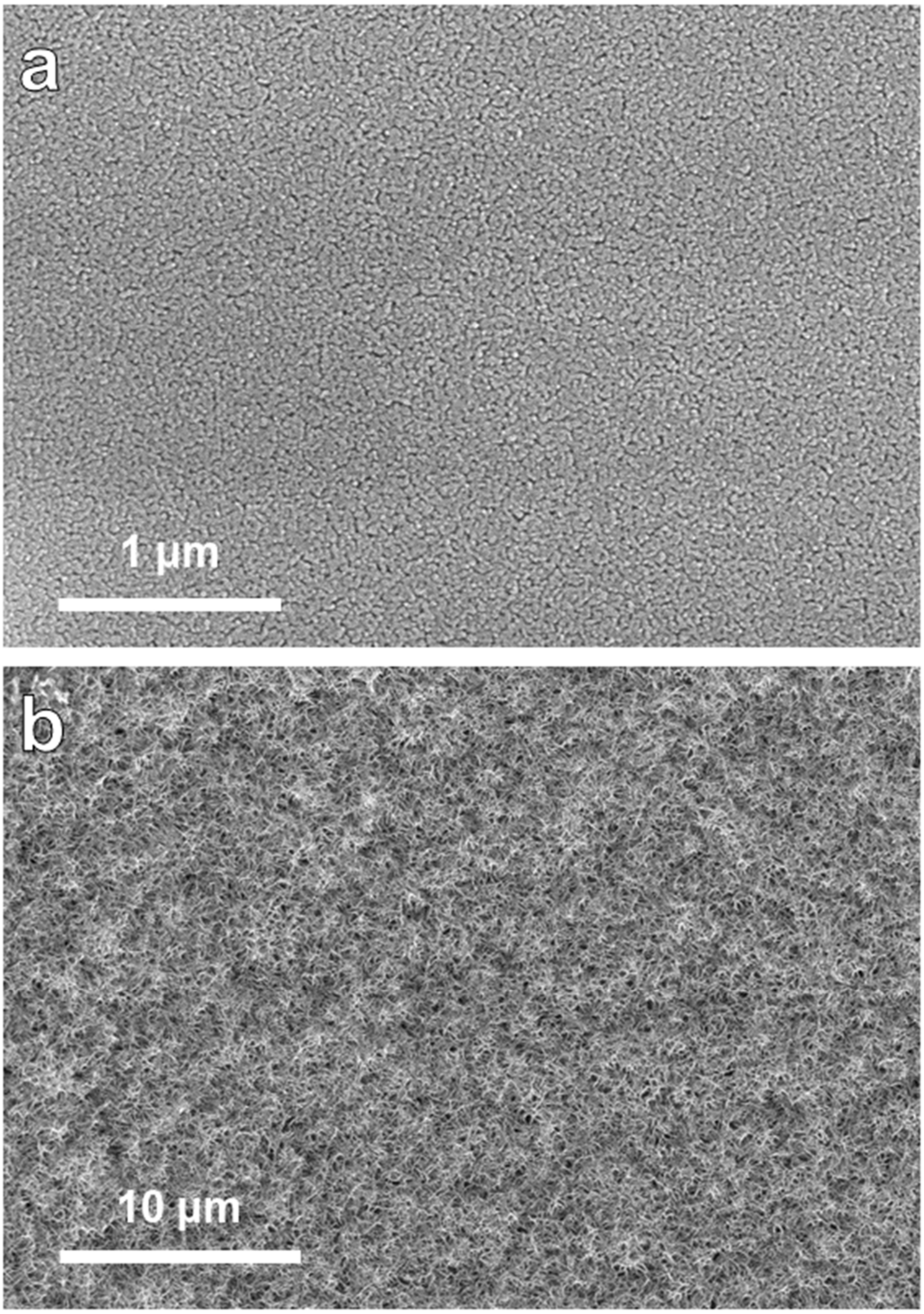

Figure S3. SEM images of a wide range of $Cu_{40}Zr_{60}$ amorphous alloy ribbon dealloyed in 0.05 M HF for 2 h (**a**) and the composite sample via deposition at -0.8 V for 30 min followed by the calcination (**b**).

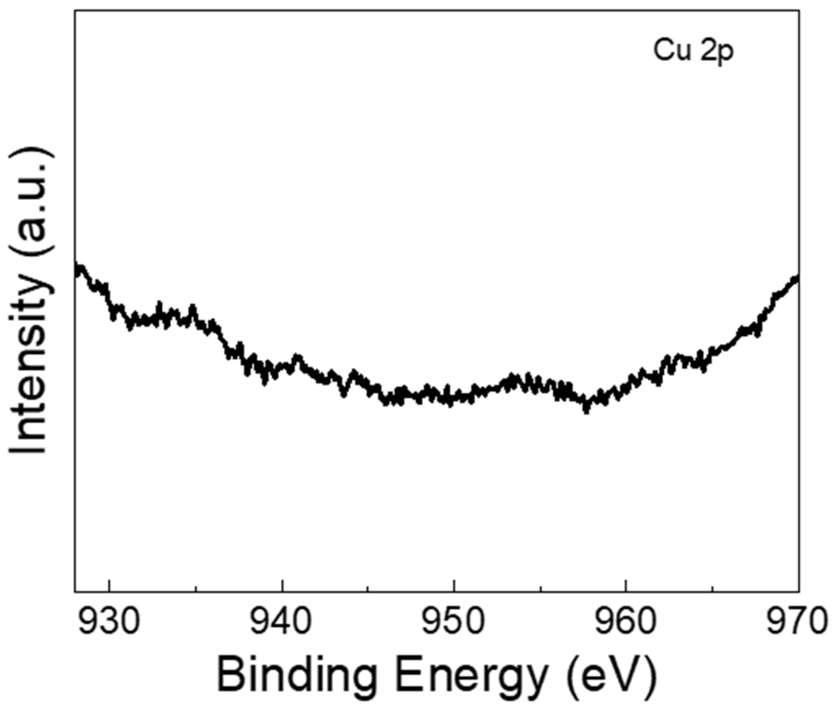

Figure S4. XPS spectrum of Cu 2p for nanopetals layer.
